# Light-Induced Electronic Band Realignment at the Metal Halide Perovskite/Monolayer MoS_2_ Heterojunction

**DOI:** 10.1021/acsami.5c02989

**Published:** 2025-05-12

**Authors:** Fengshuo Zu, Rongbin Wang, Lennart Frohloff, Nicolas Zorn-Morales, Sylke Blumstengel, Emil List-Kratochvil, Patrick Amsalem, Norbert Koch

**Affiliations:** † 28340 Helmholtz-Zentrum Berlin für Materialien und Energie GmbH, 12489 Berlin, Germany; ‡ Institut für Physik & Center for the Science of Materials Berlin, 9373 Humboldt-Universität zu Berlin, 12489 Berlin, Germany; § Institut für Chemie & Center for the Science of Materials Berlin, Humboldt-Universität zu Berlin, 12489 Berlin, Germany

**Keywords:** metal halide perovskite, monolayer MoS_2_, photoelectron spectroscopy, interfaces, electronic energy levels

## Abstract

van der Waals (vdW) heterojunctions offer many routes for advanced interface engineering toward superior optoelectronic functionality. To this end, the combination of 2D transition metal dichalcogenides (TMDCs) with metal halide perovskites has shown great potential for applications in photovoltaics and photodetectors. The electronic energy level alignment at such heterojunctions, i.e., the relative alignment of valence and conduction bands of the two materials, is crucial for their functionality, but its experimental determination is notoriously challenging. In this contribution, we determine the energy level alignment for the vdW heterojunction composed of monolayer molybdenum disulfide (ML-MoS_2_) and a triple cation-mixed halide perovskite, enabled by surface cleaning by argon cluster sputtering. This effectively removes surface contaminants from the perovskite/ML-MoS_2_ stack without causing damage, enabling direct determination of the band alignment at the interface using ultraviolet and X-ray photoelectron spectroscopy. Our results reveal a type-II band alignment at the perovskite/ML-MoS_2_ interface. Importantly, the interfacial energy levels are not fixed once the heterojunction is formed, but the MoS_2_ energy levels shift relative to those of the perovskite under 1 sun illumination compared to the dark, by up to 0.25 eV. This energy level realignment, under conditions mimicking a photovoltaic device under operation, is attributed to photogenerated electron accumulation in the ML-MoS_2_. Microscopic photoluminescence (PL) measurements reveal significant quenching of the perovskite PL signal in the heterojunction, confirming efficient charge transfer and the establishment of a type-II heterojunction. These results demonstrate a “living” heterojunction energy landscape, opening up novel avenues for engineering perovskite/TMDCs vdW heterojunctions for optoelectronic devices.

## Graphical abstract

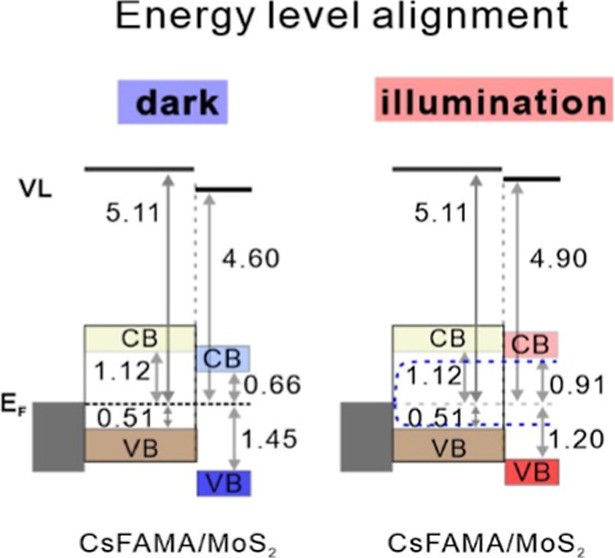

## 1 Introduction

Integration of diverse materials into heterojunctions with unique electronic and optical properties has enabled advanced interface engineering in optoelectronic devices.^1–3^ In particular, van der Waals (vdW) heterojunctions, typically involving two-dimensional (2D) materials, allow for stacking of a wide variety of materials without requiring lattice matching, thus minimizing interface states and defects that are detrimental to device performance.^3^ In this context, combining 2D transition metal dichalcogenides (TMDCs) with metal halide perovskites has shown great potential for applications in photovoltaics and photodetectors.^1,2^ Both material classes have attracted tremendous attention due to their unique optoelectronic properties. Among them, monolayer molybdenum disulfide (ML-MoS_2_) and triple cation-mixed halide perovskite both exhibit direct bandgaps,^4,5^ high optical absorption coefficients,^6,7^ and high charge carrier mobilities,^8,9^ making them ideal candidates for tailored functional vdW heterojunctions.

Significant efforts are made to incorporate TMDCs into perovskite-based solar cells, either as charge transport layer and/or as modifying layer at the interface,^1,2,10,11^ with the aim of achieving energy level matching and reduced interface nonradiative recombination and enhancing the device stability. For instance, the use of MoS_2_ as both electron (ETL) and hole transport layer (HTL) has shown enhancement in photovoltaic open-circuit voltage (*V*_oc_) and stability,^12–15^ implying a favorable energy level alignment at the perovskite/MoS_2_ interface. Tang et al.^15^ demonstrated improved hole extraction by using MoS_2_ flakes as HTL; on the other hand, Singh et al.^12^ reported the application of a thin MoS_2_ layer as ETL in perovskite-based solar cells, showing efficient charge transfer at the perovskite/MoS_2_ interface. The ambipolar transport behavior of the MoS_2_ layer implies a type-II heterojunction formation upon contact with a perovskite absorber. This is consistent with the fact that the Fermi level (*E*_F_) position of both materials within the band gap is not fixed and can be modulated by the substrate’s work function.^16,17^ Several groups have attempted to directly access the band alignment at the perovskite/MoS_2_ interface using ultraviolet photoelectron spectroscopy (UPS).^12,15,18,19^ But huge experimental challenges, such as probing the buried interface and achieving an atomically clean MoS_2_ overlayer of sufficient size for UPS analysis, have hindered the precise determination of the energy level alignment. As a result, the exact level alignment at this interface remains unknown.

In this study, we prepare a vdW heterojunction made of ML-MoS_2_ and a triple cation-mixed halide perovskite (abbreviated to CsFAMA) using a dry transfer method for the MoS_2_ monolayer. The perovskite/ML-MoS_2_ stack was cleaned by argon cluster sputtering and measured by UPS and X-ray photoelectron spectroscopy (XPS). We first demonstrate the effective use of argon cluster sputtering in removing surface contaminants without damaging the perovskite/ML-MoS_2_ stack. This surface cleaning method allows for the detection of the MoS_2_ valence band and direct access to the band alignment at the interface. Our photoemission measurements reveal a type-II band alignment at the CsFAMA/ML-MoS_2_ heterojunction. Under operando condition with 1 sun illumination, mimicking an operational photovoltaic cell, the energy levels of MoS_2_ shift by 0.25 eV relative to the perovskite, indicating a realignment of interfacial energy levels due to the accumulation of photogenerated electrons in the MoS_2_ layer, as evidenced by the filled conduction band at the Brillouin zone’s *K* point. Such energy level realignment at the atomic scale highlights the change of energy offset for electrons and has not been previously reported for perovskite/2D material interfaces. Additionally, microscopic photoluminescence (PL) measurements exhibit a strong quenching of the perovskite PL signal with the ML-MoS_2_ overlayer, confirming efficient charge transfer and the establishment of a type-II junction. Overall, the combined use of various techniques, including photoemission spectroscopy, argon cluster sputtering, and PL, has enabled a comprehensive understanding of the electronic structure of a clean monolayer MoS2 and its interfacial energy level alignment with a perovskite, which is highly relevant for optoelectronic devices based on this material system and will enable knowledge-based development of further applications.

## 2 Results and Discussion

### 2.1 Application of Argon Cluster Sputtering on the Perovskite/ML-MoS_2_ van der Waals Stack

The chemical vapor deposition (CVD)-grown ML-MoS_2_ on sapphire was initially examined by optical microscopy and Raman spectroscopy, as shown in Figure S1 in the Supporting Information. The optical image shows a continuous coverage of MoS_2_ and Raman revealed a separation of 18.9 cm^–1^ between the characteristic in-plane and out-of-plane vibrational modes, consistent with previously reported values for ML-MoS_2_.^20^ The ML-MoS_2_ layer was then transferred onto perovskite films in a N_2_-filled glovebox using the dry transfer method^21^ without involving any wet chemical processes (after contact of ML-MoS_2_ and the perovskite), which is vital for perovskite samples due to their fast decomposition in polar solvents.^22^ Details of the transfer process are described in the Experimental Details section. The use of poly­(methyl methacrylate) (PMMA) and polydimethylsiloxane (PDMS) in the transfer processes leave residues of these polymers on the ML-MoS_2_ surface, which are subsequently removed by using an argon-based gas cluster ion beam (GCIB). The use of GCIB is found effective in removing various surface contaminants (e.g., organic residues)^23–26^ and allows for highly uniform and precise surface cleaning over large areas without damaging the underlying material compared to the traditional monoatomic ion beam cleaning technique as a consequence of its significantly lower energy per atom. The argon cluster size distribution applied in this study is shown in Figure S2 and is centered at ca. 2700 argon atoms, corresponding to kinetic energy per argon atom of ca. 1.8 eV/Ar given the accelerating voltage at 5 kV.

The schematic of the CsFAMA/ML-MoS_2_ stack is displayed in Figure 1a, along with the optical image in Figure 1c and atomic force microscopy (AFM) images of pristine CsFAMA and with a MoS_2_ overlayer in Figure 1b,d, respectively. It is worth noting that the optical and AFM characterizations of the CsFAMA/ML-MoS_2_ sample were conducted after the GCIB cleaning and photoemission measurements (vide infra). The optical microscopy image demonstrates a very high surface coverage (more than 90% in the analyzed area) of ML-MoS_2_ on the CsFAMA surface, where the gray area indicates the coverage of MoS_2_.

**Figure 1:**
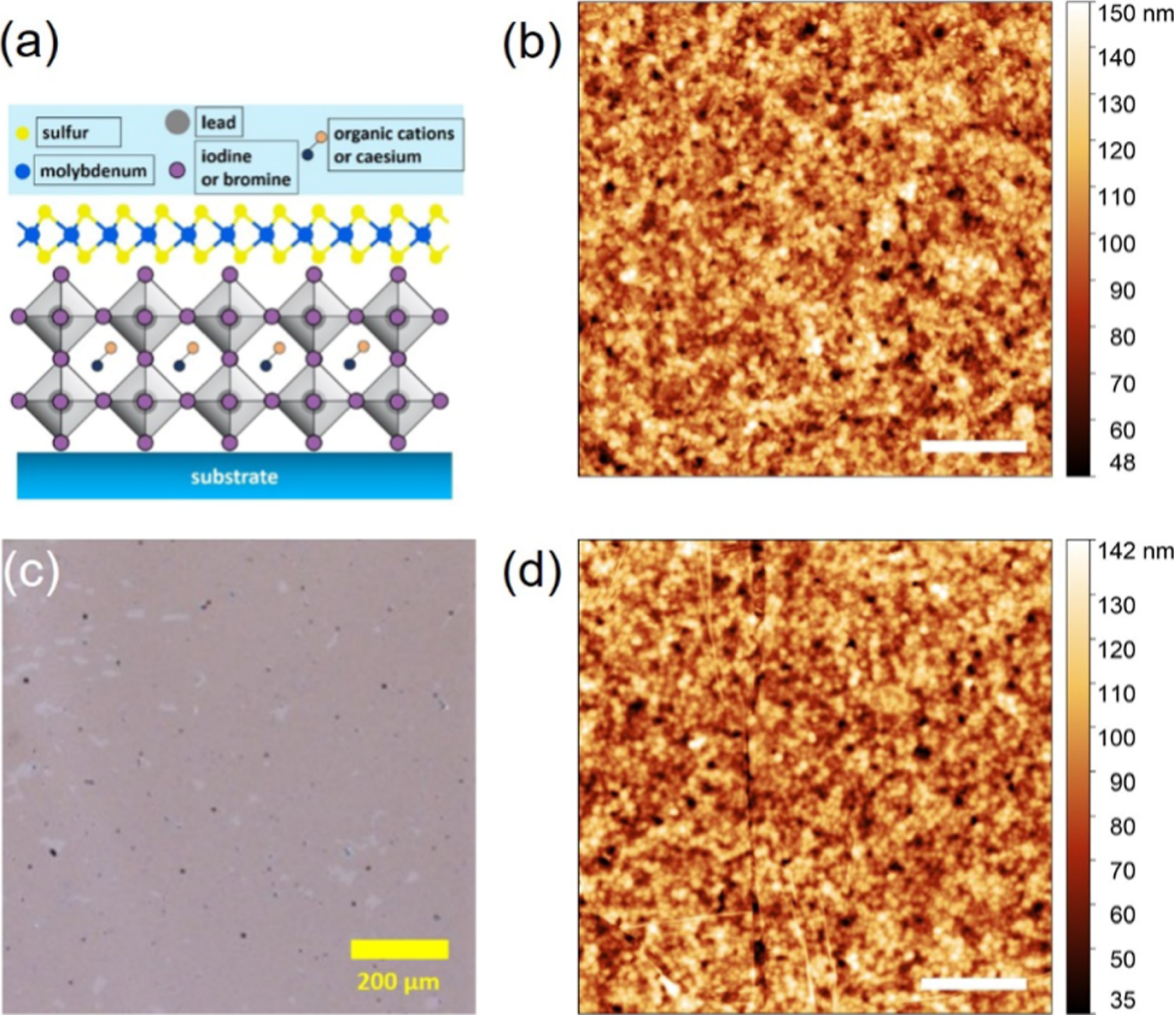
(a) Schematic diagram of the perovskite/ML-MoS_2_ stack. AFM images of the bare perovskite surface in (b) and with ML-MoS_2_ overlay in (d). (c) Optical microscopy image of the perovskite/ML-MoS_2_ stack. The black spots in Figure 1c are likely attributed to PDMS residues. AFM and optical images are taken after argon cluster sputtering cycles. Scale bar of AFM denotes 2 μm. The root mean roughness values of the bare perovskite surface and with ML-MoS_2_ overlay are 16.6 and 17.3 nm, respectively. The boundary and wrinkles in (d) are further indicated with lines as a guide to the eyes in Figure S3.

The topography image of the bare CsFAMA surface, displayed in Figure 1b, reveals a typical granular and textured perovskite surface with a relatively high surface roughness, characterized by a root mean roughness of 16.6 nm. On the other hand, the CsFAMA/ML-MoS_2_ surface in Figure 1d exhibits a globally similar topography, albeit with somewhat blurred regions and sharp boundaries indicative of the ML-MoS_2_ overlayer. This is further illustrated in Figure S3 where the coverage and boundary of ML-MoS_2_ can be readily seen. Note that although the dry transfer method demonstrated successful transfer of ML-MoS_2_ onto a rather rough perovskite surface with high coverage, the technique can lead to the formation of wrinkles, likely due to the applied pressure and/or the sudden release of strain during the transfer process.

### 2.2 Dark and Operando Energy-Level Alignment at the Perovskite/ML-MoS_2_ vdW Heterojunction

To access the band alignment of the perovskite/ML-MoS_2_ heterojunction, the CsFAMA/ML-MoS_2_ stack was incrementally exposed to an argon cluster beam with an energy distribution centered at about 1.8 eV/Ar. Following each sputtering cycle, the electronic structure and chemical composition of the sample were investigated by UPS and XPS, respectively. Figure 2a,b presents the UPS spectra of the bare CsFAMA film and of the CsFAMA/ML-MoS_2_ stack before and after multiple argon cluster sputtering cycles. Initially, the bare CsFAMA film deposited on poly­(2,3-dihydrothieno-1,4-dioxin)–poly­(styrenesulfonate) (PEDOT:PSS) exhibits a work function (Φ) of 5.11 eV, as determined from the secondary electron cutoff (SECO) in Figure 2a. The valence band (VB) onset is located at 0.51 eV with respect to the Fermi level [set to 0 eV binding energy (BE)], extrapolated on a logarithmic intensity scale due to the very low density of states near the band edge,^27,28^ as shown in Figure S4. The as-fabricated CsFAMA/ML-MoS_2_ stack exhibits no distinct features below the 3.5 eV BE VB region due to the presence of surface contaminants. Removal of these contaminants, originating from the residues of PMMA and/or PDMS left after transfer, often necessitates high-temperature annealing (above 300 °C) in ultrahigh vacuum (UHV) condition,^29,30^ which, however, cannot be performed due to the instability of perovskite materials at this temperature in vacuum. Increasing the sputtering time gradually reveals the VB features of ML-MoS_2_, with the most pronounced features (around 2–3.5 eV BE) becoming fully evident after 33 min of sputtering. Although the momentum of the VB photoelectrons from ML-MoS_2_ is averaged over all directions of the Brillouin zone due to its azimuthal disorder, photoemission spectra are dominated by the photoelectrons along the high-symmetry directions (Γ–Κ and Γ–M directions). This dominance arises from the much higher photoelectron intensity in the high-symmetry directions of 2D TMDCs.^29,31^ Thus, the VB spectra acquired at normal emission (Γ point) with the ±10° acceptance angle reveal the local VB maximum (VBM) at 1.40 eV, which is very close to the global VBM at the *K* point at 1.33 eV, as detailed in Figure 3 and consistent with the literature.^4,29^ Simultaneously, we also observe a gradual decrease in sample Φ from 4.94 to 4.72 eV after the sputtering cycles, where the small tailing toward the low kinetic energy side is attributed to the inhomogeneous spatial distribution of the surface electrostatic potential (SEP)^32^ occurring during the sputtering process, implying the composition change of the surface contaminants.

**Figure 2:**
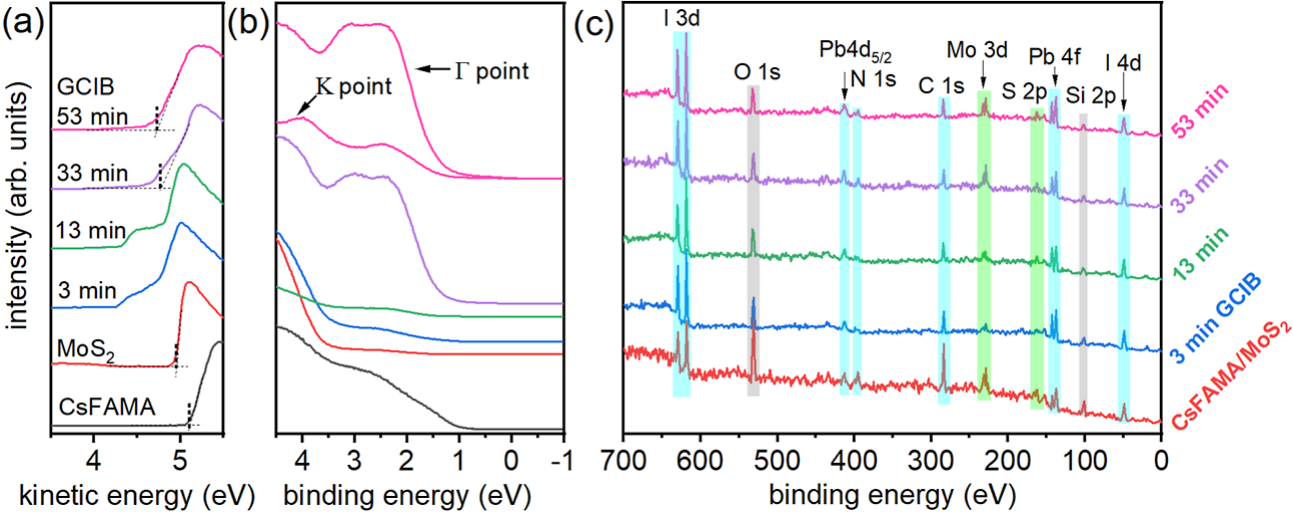
(a,b) UPS spectra and (c) XPS survey of the bare CsFAMA and the CsFAMA/ML-MoS_2_ stack after argon cluster sputtering for various durations. Evolution of SECO in (a) and valence band in (b) upon argon cluster sputtering. The contribution of various elements from XPS survey is indicated by different colors: light blue, light green, and gray denote the contributions from perovskite, MoS_2_, and PDMS residue, respectively. Each XPS survey spectrum is normalized with respect to I 3d core levels in order to contrast the relative change of each core level.

**Figure 3:**
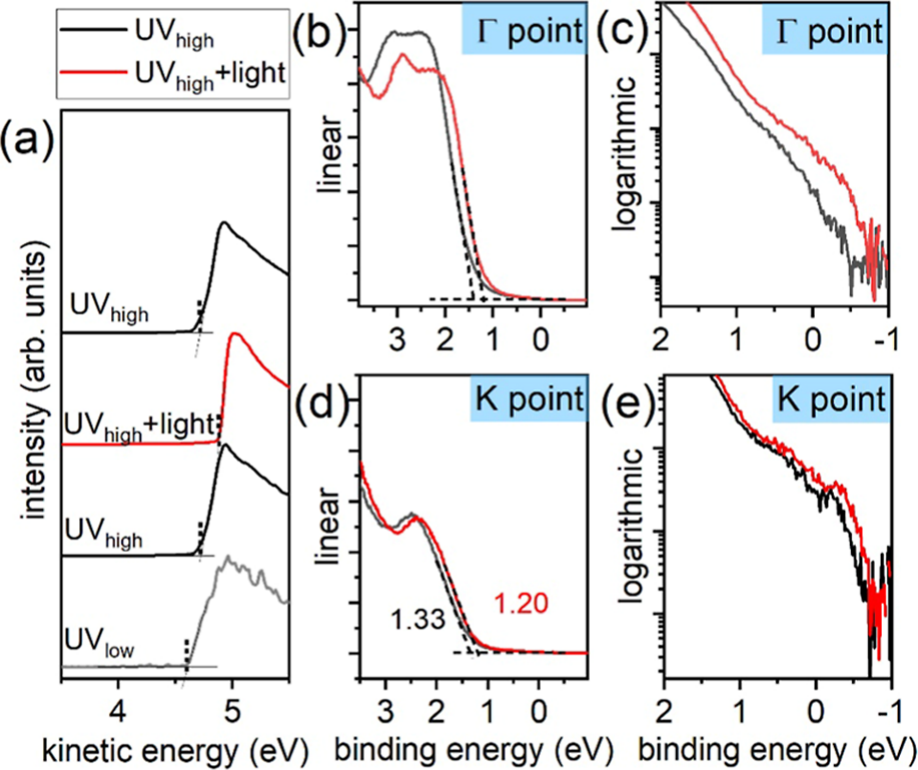
UPS spectra of the CsFAMA/ML-MoS_2_ stack measured under various UV flux and 1 sun illumination. (a) SECO; valence bands measured at the Γ point in (b,c) and *K* points in (d,e).

The chemical composition characterization by XPS was also performed after each sputtering cycle, as shown in Figure 2c. The survey spectra show a clear increase in the contributions of the Mo 3d and S 2p core levels, compared to the surface contaminant-related O 1s and Si 2p core levels. For instance, the photoelectron intensity ratio of Mo 3d_5/2_ to O 1s increases significantly by approximately 2.2 times after 33 min of sputtering and remains unchanged after 53 min. This increase is also accompanied by a significant rise in the photoelectron intensity of the perovskite core levels (I 3d and Pb 4f), signifying an efficient removal of surface contaminants. The presence of oxygen and silicon signals can be ascribed to the residue of PDMS used as the stamp material in the dry transfer process. To highlight the importance of minimizing the argon atom kinetic energy, we also performed argon cluster sputtering at a higher energy of approximately 3.6 eV/Ar on ML MoS_2_ transferred onto a silicon wafer, as shown in Figure S5. It can be clearly seen that significant degradation already occurs within just 10 min of sputtering, including the formation of metallic molybdenum, substantial sulfur loss, and the emergence of density of states near the Fermi level at 0 eV binding energy.

To reliably establish the band alignment at the perovskite/ML-MoS_2_ interface, the impact of photoexcitation (as needed for creating the photoelectrons) on the energy level alignment must be carefully examined. It was recently demonstrated that the energy levels at metal halide perovskite-related heterojunctions can realign significantly due to the redistribution of photogenerated charge carriers.^33–35^ Thus, additional UPS measurements on the sample stack under varied UV flux and additional white light (equivalent to 1 sun intensity) were performed, as shown in Figure 3. As seen from the SECO in Figure 3a, UV excitation already leads to an increase of sample SEP from 4.60 to 4.72 eV when measured under low and high UV flux (approximately 100 times difference in UV flux), respectively. Under additional 1 sun illumination, the sample SEP further increases to 4.90 eV and returns to 4.72 eV under high UV flux but without white light. Parallel shifts of the valence bands at both Γ and *K* points are observed, exhibiting nearly equivalent changes toward lower BE upon 1 sun illumination. The global VBM at the *K* point shifts from 1.33 to 1.20 eV, as determined from the linear extrapolation of the photoelectrons. It is worth mentioning that the valence band scans were acquired under high UV flux to ensure a high signal-to-noise ratio. Given the shift of the SEP by 0.12 eV solely due to UV excitation, the global VBM in dark (referring to the electronic ground state) is then extrapolated to 1.45 eV. Notably, a small peak on the logarithmic intensity scale appears near the Fermi level at the *K* point, which also rigidly shifts toward lower BE upon illumination. This peak is ascribed to the partially filled global conduction band minimum at the *K* point, as often observed for natively n-doped MoS_2_,^36^ and it is essentially absent in the VB spectra at the Γ point. The fact that the feature at the *K* point is slightly positioned above the Fermi level when measured under high UV flux is consistent with the shift of the SEP caused by high UV flux. As expected, the photoexcitation-driven shifts of the ML-MoS_2_ energy levels are also evident from the Mo 3d core levels in Figure S6, which displays similar shifts toward lower BE simply due to increased X-ray flux. In contrast, the perovskite core levels remain constant upon increasing the X-ray anode power, indicating fixed energy levels of perovskite upon photoexcitation. It is worth noting that we do not observe the appearance of reduced Mo states at lower BE side after argon cluster sputtering, indicating the absence of ion bombardment-induced degradation in MoS_2_.^37^ As a side note, we remark that the overlayer of ML-MoS_2_ on the perovskite surface is expected to significantly slow down the photodecomposition process of the perovskite in UHV, enabling prolonged and detailed photoemission measurements before photodegradation occurs.^38^

Above observations clearly demonstrate a realignment of the ML-MoS_2_ energy levels with respect to those of the perovskite substrate, i.e., a change of the interfacial energy offsets upon photoexcitation. The energy level diagrams as determined from the PES measurements are presented in Figure 4. A type-II alignment at the perovskite/ML-MoS_2_ heterojunction is present with an energy offset of about 0.46 eV for electron extraction as given by the energy offset of the conduction band minima of both materials. Bandgap values of 1.63 eV^39^ for CsFAMA perovskite and 2.11 eV^16^ for ML-MoS_2_ are taken from literature. Under illumination, the charge selective nature of the interface results in an unbalanced charge distribution at the vdW junction, where electrons accumulate in the MoS_2_, causing a shift of its energy levels relative to the perovskite substrate.

**Figure 4:**
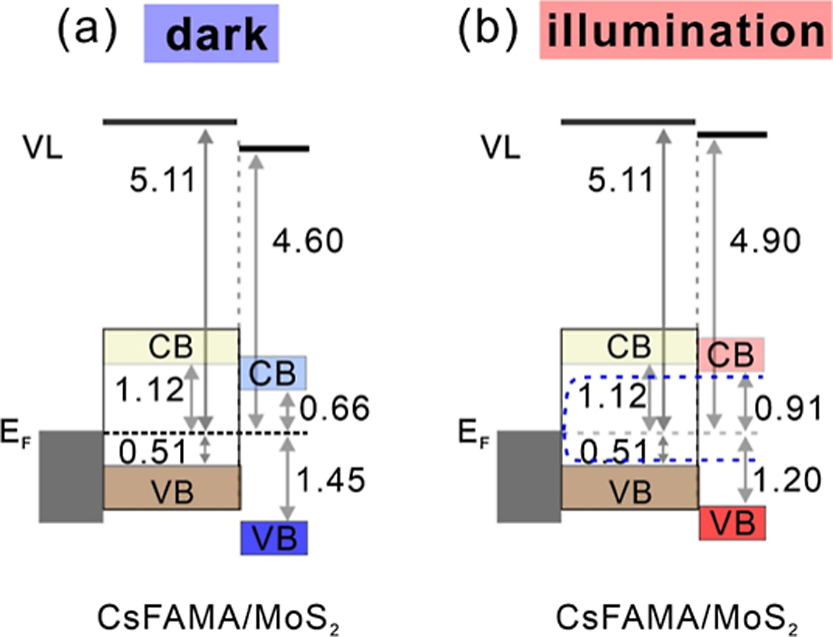
Schematic energy level diagrams of the CsFAMA/ML-MoS_2_ stack (a) in dark and under (b) 1 sun illumination. The global VBM of ML-MoS_2_ is extracted at 1.45 eV in dark (referring to its electronic ground state), given the UV induced shift in the SEP. Bandgap values of 1.63 eV^39^ for CsFAMA perovskite and 2.11 eV^16^ for ML-MoS_2_ are taken from literature. All values are referred to the *E*_F_ and in unit of eV. VB, CB, and VL denote valence band, conduction band, and vacuum level, respectively. Blue dashed lines in (b) indicate the quasi-Fermi levels upon photoexcitation.

To further corroborate the establishment of a type-II heterojunction at the perovskite/ML-MoS_2_ interface, steady-state PL measurements with a lateral resolution of ca. 1 μm (determined by the laser diameter) were conducted on the same CsFAMA/ML-MoS_2_ stack in a N_2_-filled environment, as shown in Figure 5. To ensure the reproducibility and reliability of the PL intensity measurements on the perovskite sample, multiple sampling points were collected on the perovskite-only areas (found directly from visual inspection by optical microscopy), as shown in Figure S7. These measurement points exhibited a consistent PL intensity across several perovskite domains. In comparison, the ML-MoS_2_ covered region showed a significant PL quenching compared to the bare perovskite region, clearly indicating efficient charge transfer to the ML-MoS_2_ upon photoexcitation, in agreement with the formation of a type-II heterojunction.

**Figure 5:**
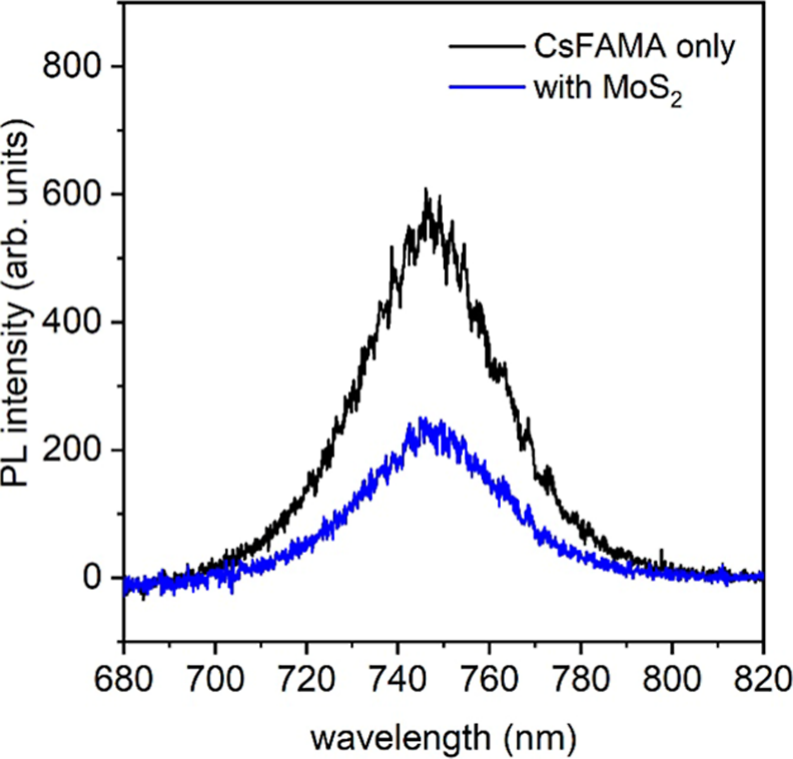
Microscopic PL measurements on the bare CsFAMA domain and with ML-MoS_2_ overlay.

## 3 Conclusion

This study demonstrates the fabrication of a vdW heterojunction composed of ML-MoS_2_ and triple cation perovskite using a dry transfer method, post-transfer cleaning by GCIB sputtering, and its reliable characterization by photoemission spectroscopy. The use of GCIB with 1.8 eV/Ar is found to be effective in removing surface contaminants without damaging the CsFAMA/ML-MoS_2_ stack, enabling the detection of the MoS_2_ VB and establishment of the band alignment at the interface. Photoemission measurements reveal a type-II alignment at the CsFAMA/ML-MoS_2_ heterojunction. However, the band alignment is not fixed upon heterojunction formation, but the MoS_2_ energy levels shift by 0.25 eV relative to those of the perovskite upon 1 sun illumination. This shift reveals a realignment of the interfacial energy levels due to the accumulation of photogenerated electrons in the MoS_2_ layer, as evidenced by the partially filled conduction band at the *K* point. Additionally, microscopic PL showed a strong quenching of the perovskite PL signal with the ML-MoS2 overlayer, further confirming efficient charge transfer and the establishment of a type-II junction. These results highlight the potential of perovskite/TMDCs heterojunctions for enhancing the performance of optoelectronic devices and disclose the light-induced band realignment as an important factor for material selection and functionality.

## 4 Experimental Details

### 4.1 Sample Preparation

#### 4.1.1 Substrates

Indium-tin-oxide (ITO) substrates were cleaned with acetone, deionized water, and isopropanol by sonication for 10 min in each solution. After UV–ozone treatment for 10 min, the ITO substrates were spin-coated with poly­(3,4-ethylenedioxythiophene):polystyrenesulfonate (PEDOT:PSS, AI 4083) at a speed of 3000 rpm for 60 s, followed by annealing at 150 °C for 15 min in a N_2_-filled glovebox.

#### 4.1.2 Perovskite Samples

The triple cation-mixed halide perovskite solution was prepared according to ref 35. In short, 1.2 M formamidinium lead iodide (FAPbI_3_) and methylammonium lead bromide (MAPbBr_3_) perovskite solutions in a particular volume ratio of 83:17 results in a nominal perovskite stoichiometry of Cs_0.05_(FA_0.83_MA_0.17_)_0.95_Pb­(I_0.83_Br_0.17_)_3_ (abbreviated as CsFAMA). The CsFAMA films were prepared in the N_2_-filled glovebox by spin-coating the precursor onto ITO/PEDOT:PSS substrates at 4000 rpm for 30 s, and 0.2 mL of ethyl acetate was dropped onto the spinning substrate at a delay time of 8 s after the start of the spin-coating. Then, the perovskite films were annealed at 100 °C for 10 min.

#### 4.1.3 Monolayer MoS_2_ by Chemical Vapor Deposition

Large-scale MoS_2_ monolayers were grown by the CVD method. Briefly, 0.015 M NaMoO_4_·2H_2_O as the Mo source was spin-coated on the precleaned sapphire substrate. The substrate was then placed at the center of the quartz tube, and sufficient sulfur powder was placed upstream. The quartz tube was then pumped down to ∼10^–2^ mbar and subsequently purged with Ar gas up to atmospheric condition. Next, the oven was heated up to 800 °C in 50 min and maintained at this temperature for 10 min for MoS_2_ growth. Ar gas with a purity of 99.99% was used as the carrier gas at 60 standard cubic centimeters per minute (sccm).

### 4.2 Transfer of MoS_2_ onto Perovskite Substrates

A multistep process is necessary to obtain a clean MoS_2_ layer on top of the CsFAMA perovskite surface, as illustrated in Figure 6. In short, (1) a PMMA film was spin-coated onto the as-grown ML-MoS_2_ on a sapphire substrate; (2) the sapphire/MoS_2_/PMMA stack was immersed in a KOH solution to separate the MoS_2_ from sapphire, followed by rinsing the PMMA/MoS2 stack in deionized water; (3) the PMMA/MoS2 stack was transferred onto Si wafer coated with a PVA (poly­(vinyl alcohol)) layer; (4) acetone vapor was used to remove the PMMA layer, after which PDMS was applied onto the Si/PVA/MoS_2_ stack; (5) deionized water was used to dissolve the PVA, separating the PDMS/MoS_2_ stack from Si wafer, followed by immersing of the PDMS/MoS_2_ stack in deionized water for 3 times to remove the PVA residue; (6) the PDMS/MoS_2_ stack was transferred onto the perovskite surface using the dry transfer method; (7) the argon-based GCIB was employed to remove the PDMS and/or PMMA residues. Note that the inclusion of a PVA layer is crucial for successfully detaching the PMMA/MoS_2_ stack from the Si wafer.

**Figure 6:**
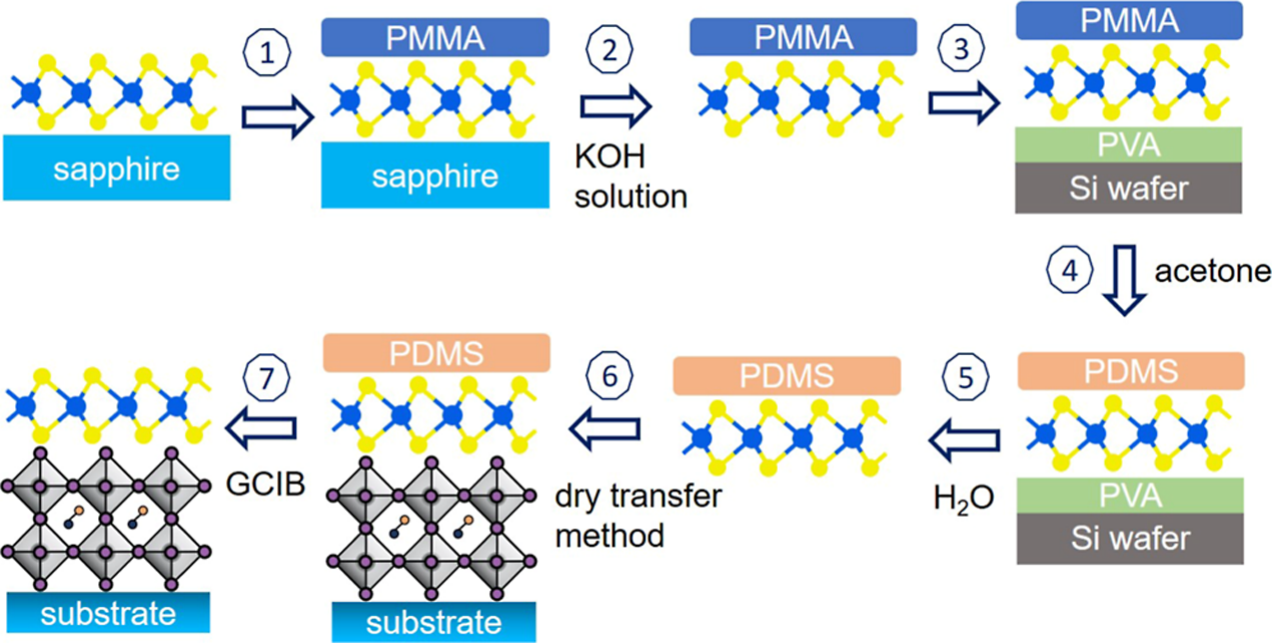
Schematic drawing of the main steps for assembly of the CsFAMA/ML-MoS_2_ van der Waals stack.

#### 4.2.1 Gas Cluster Ion Beam

Argon-based GCIB sputtering was performed using the GCIB 10S by Ionoptika, which allows for controlling of the cluster size and kinetic energy. The sputter area of ca. 67 mm^2^ is determined from the built-in sample current imaging system. The cluster size distribution employed in this study is directly measured by time-of-flight mass spectroscopy, as shown in Figure S2, resulting in a kinetic energy per argon atom at ca. 1.8 eV/Ar at peak maximum. The GCIB chamber is integrated into an UHV cluster system, which interconnects with the photoemission spectroscopy setup.

#### 4.2.2 Photoemission Spectroscopy

Photoemission measurements were conducted using a hemispherical energy analyzer (PREVAC EA15) at room temperature in the UHV cluster chamber (base pressure of 1 × 10^–9^ mbar). A monochromatized helium discharge lamp (PREVAC, photon energy of 21.22 eV) and monochromatized Al Kα radiation (PREVAC, photon energy of 1486.6 eV) generated from a twin-anode X-ray source were employed as excitation sources. Valence band measurements were conducted at normal emission and at 45° with respect to the sample normal with an acceptance angle of ±10°. SECO spectra were acquired with a sample bias of −10 V. The instrument energy resolution was set at 120 meV for UPS and 650 meV for XPS.

#### 4.2.3 Microscopic Photoluminescence Measurements

Microscopic PL measurements were conducted with a home-built PL setup using a *cw* laser diode emitting at 2.21 eV (PicoQuant) as the excitation source. An Acton SpectraPro 2500i spectrograph and a liquid-nitrogen-cooled CCD (Acton SPEC-10:100) were used as the spectrometer. The laser spot diameter was 1 μm with a power of 0.1 μW.

## Supplementary Material



## Notes

Optical microscopy image and Raman spectroscopy; argon cluster size distribution; AFM images; valence band spectrum of CsFAMA; UPS spectra of a ML MoS_2_ under argon cluster sputtering at ca. 3.6 eV/Ar; high-resolution core levels spectra; microscopic PL spectra; and valence band spectra of a ML MoS_2_ on a polycrystalline gold film (PDF)
