# Supplementary material for: Light-Induced Electronic Band Realignment at the Metal Halide Perovskite/Monolayer MoS2 Heterojunction
Source: ACS Appl Mater Interfaces. 2025 May 12;17(20):30251–8. doi: 10.1021/acsami.5c02989 (PMC12100639; doi:10.1021/acsami.5c02989)
Supplement: Supplementary file 1 [file am5c02989_si_001.pdf]

Supporting Information

Light-induced electronic band realignment at the metal halide perovskite/monolayer MoS_2 heterojunction

Fengshuo Zu,[1*] Rongbin Wang,[2] Lennart Frohloff,[2] Nicolas Zorn-Morales,[2] Sylke Blumstengel,[2] Emil List-Kratochvil,[1,2,3] Patrick Amsalem,[2] Norbert Koch[1,2*]

[1] Helmholtz-Zentrum Berlin für Materialien und Energie GmbH, 12489 Berlin, Germany.

[2] Institut für Physik & Center for the Science of Materials Berlin, Humboldt-Universität zu Berlin, 12489 Berlin, Germany.

[3] Institut für Chemie & Center for the Science of Materials Berlin, Humboldt-Universität zu Berlin, 12489 Berlin, Germany.

* Corresponding Authors: fengshuo.zu@physik.hu-berlin.de; norbert.koch@physik.hu-berlin.de

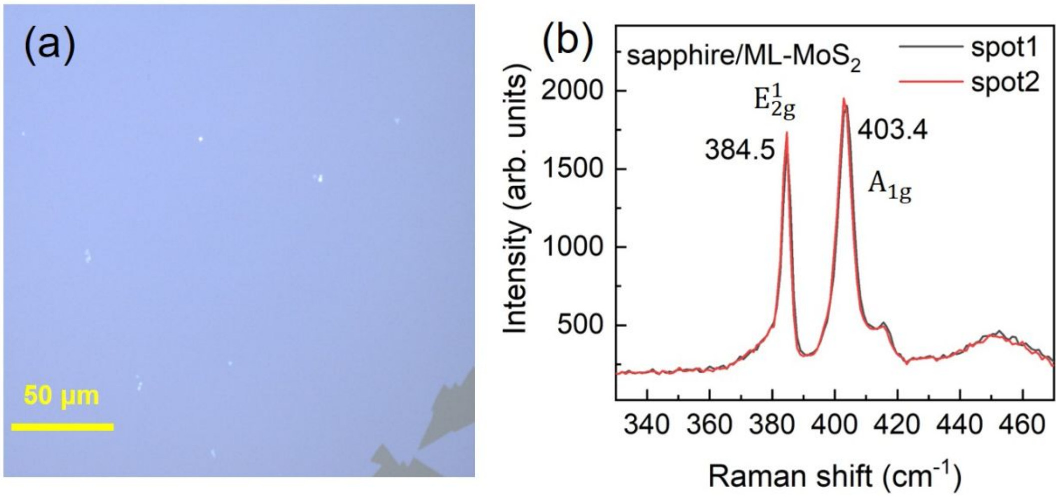

Figure S1. (a) Optical microscopy image and (b) Raman spectroscopy of ML-MoS_2 on sapphire. In order to contrast the presence of MoS_2, the optical image was taken at the location where the substrate can be seen (on the bottom-right corner). The characteristic in-plane E_{2g}^{1} and out-of-plane A_{1g} vibration modes are separated by 18.9 cm^{-1}.

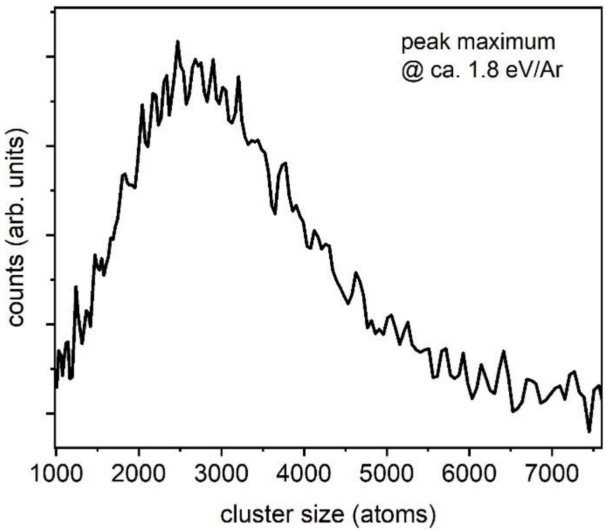

Figure S2. Argon cluster size distribution with an accelerating voltage of 5 kV determined by time-of-flight mass spectroscopy.

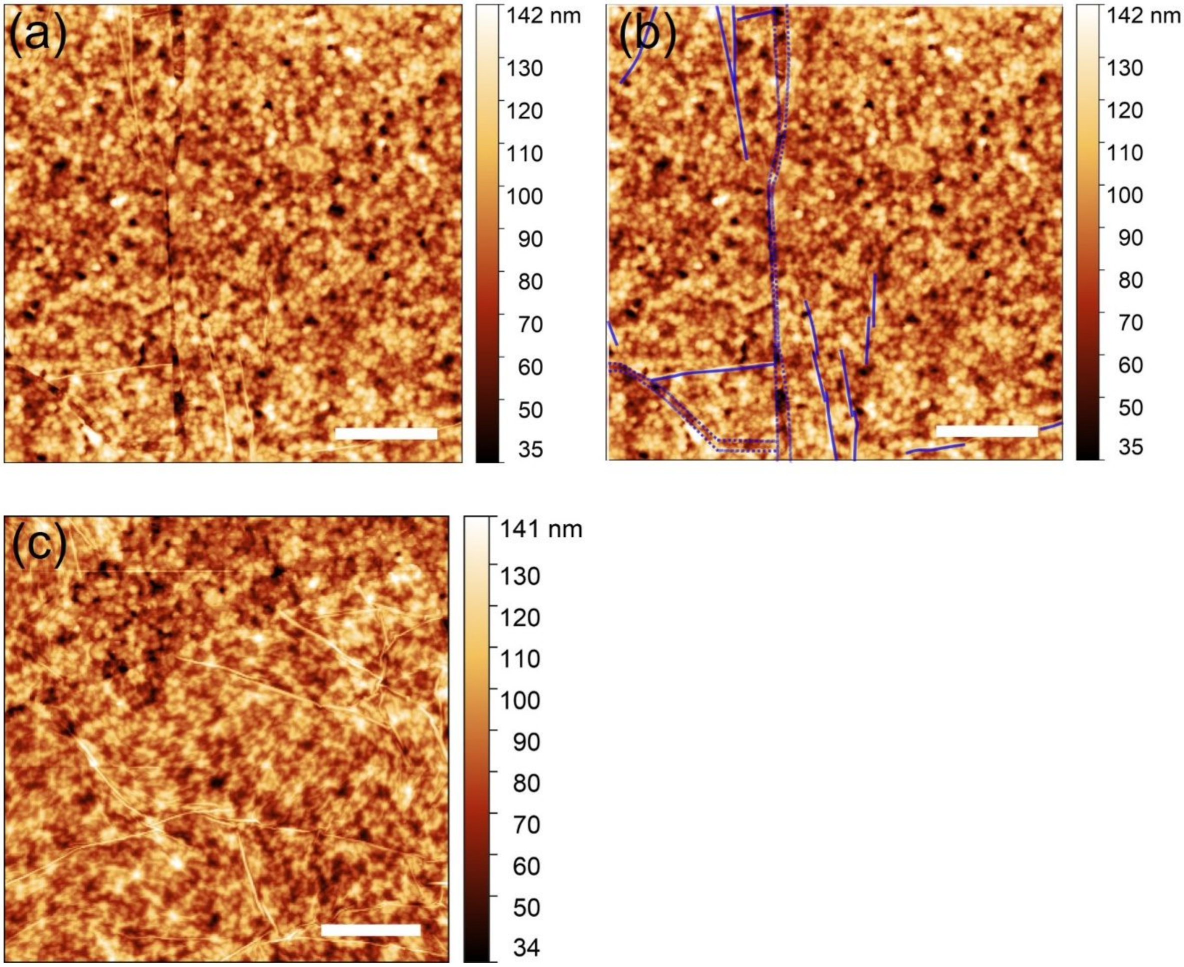

Figure S3. Atomic force microscopy images of the CsFAMA/ML-MoS_2 stack as shown in Figure 1d in the main text: (a) as-acquired and (b) with solid and dashed blue lines marked indicating the winkles and boundary, respectively. (c) in a region where a sharp boundary of ML-MoS_2 in contrast to the perovskite can be clearly seen. Scale bar denotes 2 μm.

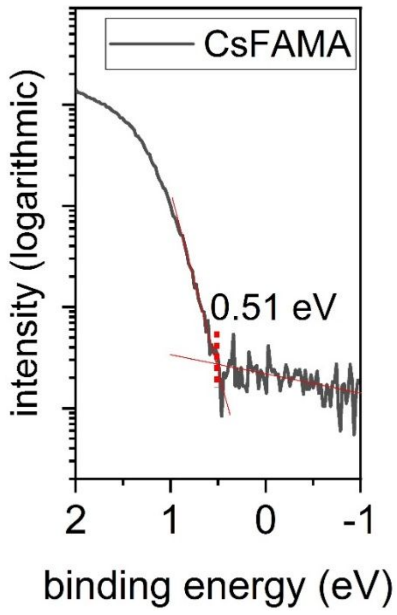

Figure S4. Valence band spectrum of the CsFAMA perovskite deposited on PEDOT:PSS on a logarithmic intensity scale.

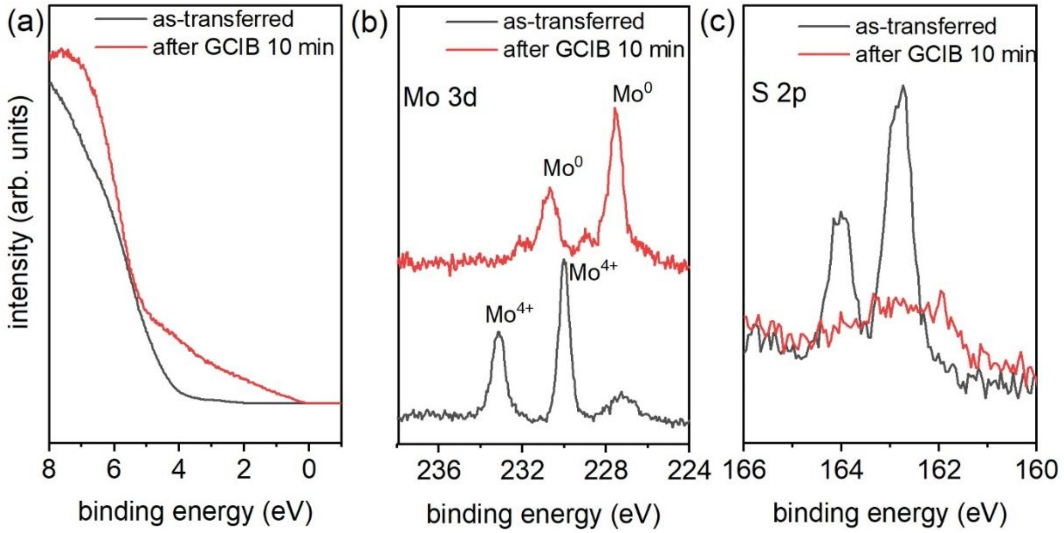

Figure S5: (a) Valence band, (b) Mo 3d and (c) S 2p core levels spectra of a ML MoS_2 transferred onto a silicon wafer before and after argon cluster sputtering at ca. 3.6 eV/Ar for 10 minutes.

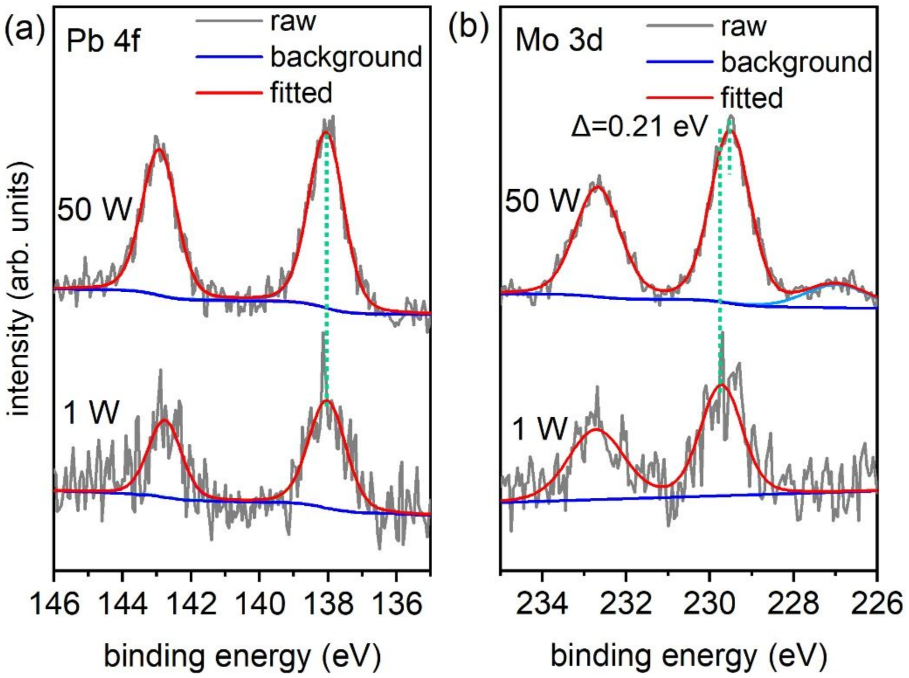

Figure S6. High resolution (a) Pb 4f and (b) Mo 3d core levels spectra of the CsFAMA/ML-MoS_2 stack measured under 1 W and 50 W X-ray anode power.

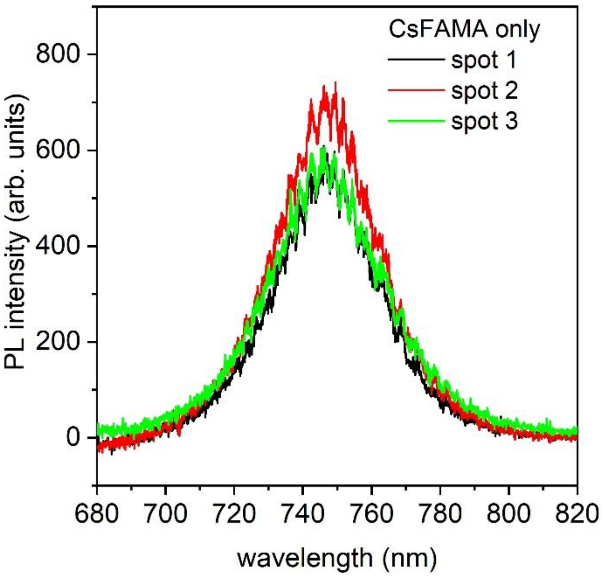

Figure S7. Microscopic PL measurements on bare CsFAMA.

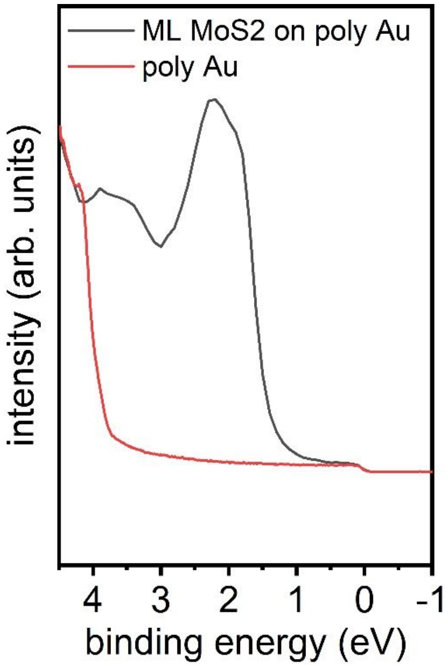

Figure S8. Angle-integrated (along K-Γ-M) UPS spectrum of a monolayer MoS_2 on a polycrystalline gold substrate. The sample was annealed at 350 °C for 8 hours before UPS acquisition.
